# simPIC:flexible simulation of paired-insertion counts for single-cell ATAC sequencing data

**DOI:** 10.1101/2025.09.21.676689

**Published:** 2025-09-23

**Authors:** Sagrika Chugh, Heejung Shim, Davis J. McCarthy

**Affiliations:** 1 Bioinformatics and Cellular Genomics, St. Vincent’s Institute of Medical Research, 9 Princes Street, Fitzroy, 3065, Victoria, Australia.; 2 School of Mathematics and Statistics, Faculty of Science, University of Melbourne, Grattan Street, Parkville, 3010, Victoria, Australia.; 3 Faculty of Medicine, Dentistry and Health Sciences, University of Melbourne, Grattan Street, Parkville, 3010, Victoria, Australia.

**Keywords:** single-cell ATAC-seq, single-cell genomics, software package, simulation

## Abstract

Single-cell Assay for Transposase Accessible Chromatin (scATAC-seq) is increasingly used at population scale to study how genetic variation shapes chromatin accessibility across diverse cell types. This widespread adoption of the assay has created a need for computational methods that can handle complex biological and technical variation. Yet method development is limited by the lack of flexible simulation tools with known ground truth. Here, we present simPIC, a simulation framework for generating realistic single-cell ATAC-seq data across individuals and cell types. simPIC supports both population-scale and single-individual simulations, with the ability to model cell groups, batch effects, and genotype-dependent variation in accessibility. These features enable realistic benchmarking for tasks such as chromatin accessibility quantitative trait locus (caQTL) mapping. simPIC generates data that closely match real datasets and better captures inter-individual and experimental variation compared to existing tools.

## 1 Background

Single-cell Assay for Transposase-Accessible Chromatin using sequencing (scATAC-seq) has emerged as a powerful technique for profiling chromatin accessibility at single-cell resolution. By employing the Tn5 transposase to preferentially insert sequencing adapters into accessible genomic regions, scATAC-seq enables detailed mapping of cell-specific regulatory landscapes. This approach provides critical insights into the interplay between chromatin structure and gene expression [1, 2]. Despite its advantages, scATAC-seq data are inherently sparse and noisy. Consequently, most analytical strategies to date have focused on clustering cells into discrete groups or aggregating accessibility signals across genomic peaks. While effective in many contexts, these approaches may obscure the underlying biological variability present at the single-cell level. New data analysis approaches are needed, but relative to the rapid advances in experimental techniques, the computational infrastructure for scATAC-seq data analysis remains underdeveloped [3]. Notably, flexible and biologically realistic simulation frameworks are lacking, which constrains rigorous evaluation and benchmarking of novel computational methods.

Simulation is fundamental for benchmarking analytical tools, optimizing experimental designs, and developing new computational approaches by providing controlled settings with known ground truth. In single-cell RNA sequencing (scRNA-seq), simulation tools have advanced notably to name just two: Splatter offers flexible models to simulate data for cells from a single individual [4], while splatPop extends the Splatter framework to population-scale data by modelling variation across individuals, cell types, and experimental conditions [5]. However, simulation frameworks for scATAC-seq have not kept pace. Current scATAC-seq simulators, including SCAN-ATAC-Sim [6], simATAC [7], EpiAnno [8], scMultiSim [9], simCAS [10], and scDesign3 [11] exhibit limitations that restrict their generalizability and biological realism. For example, SCAN-ATAC-Sim relies on bulk chromatin accessibility data, which limits its ability to capture single-cell resolution features, whereas EpiAnno simulations depend on prior knowledge of highly accessible peaks. scDesign3 requires high-quality cell type annotations, and scMultiSim depends heavily on real datasets for cell state assignment. While simATAC and simCAS simulate discrete cell types, they have notable drawbacks. simATAC generates bin-by-cell count matrices that inadequately represent peak-centric regulatory architecture and produces pseudo-bulk profiles due to its inability to model sparsity effectively [8, 10]. simCAS, implemented in Python, employs an embedding-based strategy to generate peak-by-cell matrices that preserve key data properties but demands extensive parameter tuning across multiple data attributes, resulting in considerable computational complexity. Critically, to our knowledge, none of these tools incorporate genetic effects such as chromatin accessibility quantitative trait loci (caQTLs), limiting their applicability in population-scale studies where genetic variation between individuals significantly shapes chromatin accessibility landscapes.

To address the need for realistic and efficient simulation of single-cell chromatin accessibility data, we developed simPIC, an R/Bioconductor package that generates peak-by-cell count matrices based on Paired Insertion Counting (PIC), a recently introduced standardized approach for quantifying scATAC-seq signal [12]. simPIC faithfully replicates key characteristics of real scATAC-seq datasets, including library size distributions (i.e., the variability in total fragments sequenced per cell, which reflects sequencing depth and technical noise), peak means (the average accessibility per genomic region, capturing the inherent activity across regulatory elements), and cell sparsity (the high proportion of zero counts, driven by biological and technical limitations). In addition to simulating distinct, homogeneous cell populations, simPIC supports complex experimental designs and sources of variation, including multiple cell types and batch effects. To model genetic heterogeneity and known chromatin accessibility QTLs (caQTLs), simPIC integrates seamlessly with the splatPop framework [5], thereby extending its application to chromatin accessibility data. This integration is crucial for mimicking the multidimensional heterogeneity present in real single-cell studies, where technical artifacts, diverse cellular identities, and inter-individual genetic variation all shape the observed chromatin landscape [13]. simPIC is freely available through Bioconductor, providing researchers with a flexible, scalable, and biologically grounded tool for simulating realistic scATAC-seq datasets.

## 2 Results

### 2.1 The simPIC model framework

**Fig. 1:**
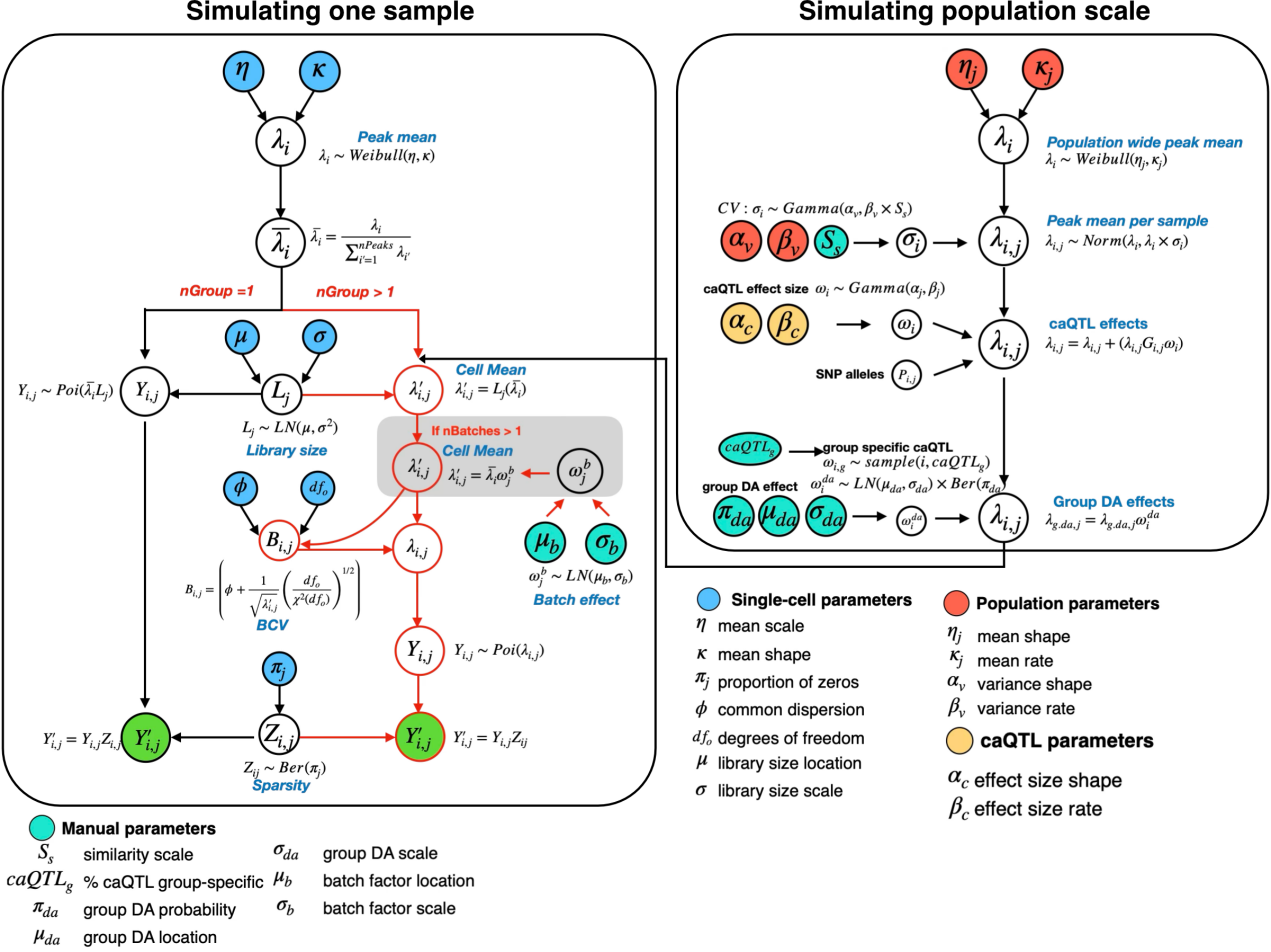
Graphical model representation of the simPIC simulation framework. Input parameters estimated from real data are indicated in blue, population parameters are indicated in orange, cyan represents manual parameters. Green circle indicates the final output, the simulated peak-by-cell matrix. Simulating population-scale data is achieved by integrating with splatPop. Abbreviations used: (*LN*)- lognormal,(*Ber*)- Bernoulli, (*Poi*)- Poisson distribution.

We designed simPIC as a modular simulation framework to generate synthetic single-cell ATAC-seq datasets that capture the key biological and technical features observed in real experiments. Implemented as an R/Bioconductor package, simPIC flexibly supports a range of experimental designs, from single-sample simulations to population-scale studies. The framework comprises two main components: (a) simulating one or more cell types within a single sample, optionally across multiple experimental batches; and (b) simulating population-scale data by incorporating genetic variation across individuals (Figure 1). The output is returned as a SingleCellExperiment object, enabling easy integration with downstream tools. To support model evaluation and calibration, simPIC includes the simPICcompare() function, which produces diagnostic plots comparing real and simulated datasets across key quality metrics such as library size distributions, peak-level accessibility, and sparsity. This modular design allows users to simulate tailored datasets that reflect realistic chromatin accessibility landscapes and biological heterogeneity for benchmarking, method development, and hypothesis testing.

#### 2.1.1 simPIC accurately reproduces single-cell accessibility profiles for individual cell type

We first evaluated the ability of simPIC to simulate scATAC-seq data for a single cell type, representing a foundational use case with minimal complexity. Using six publicly available datasets—PBMC5k, PBMC10k, Satpathy et al [14], Liver [15], Cusanovich et al [16], and Fly brain [17]. We benchmarked simPIC’s two peak modeling strategies—simPIC gamma (simPIC(g)) and simPIC weibull (simPIC(w))—against the pseudo-cell type mode of simCAS and real data. For each cell type from the above studies, we simulated data using the same number of cells as in the corresponding real dataset. To reduce noise and maintain comparability, only peaks observed in at least 1% of cells were retained; no cell-level filtering or quality control was applied. This setup allowed for rigorous testing under a high-noise regime.

**Fig. 2:**
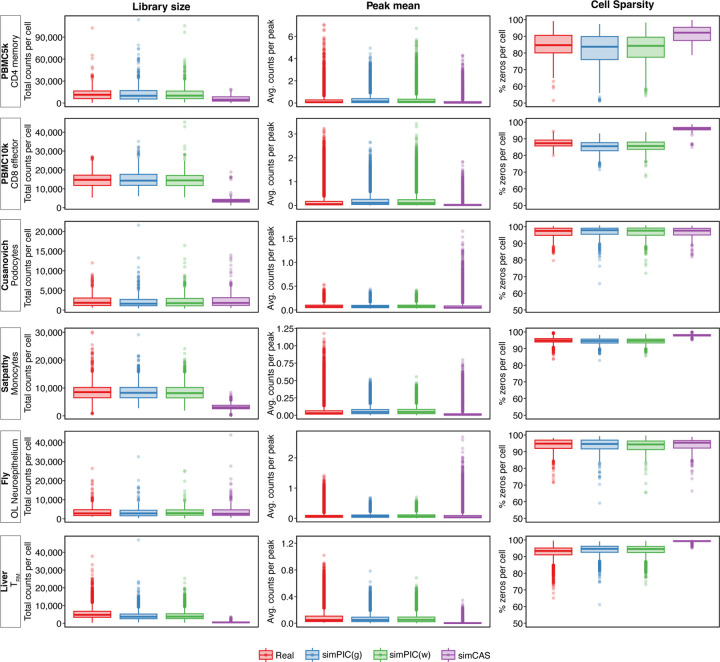
Comparison between library size, peak means and cell sparsity distributions across PBMC5k, PBMC10k, Cusanovich, Satpathy, Fly and Liver datasets generated by simPIC and simCAS. Box plots represent library size, peak means and cell sparsity, with colours denoting real data (red), Simulated data based on gamma-modelled peak means (blue) and Weibull-modelled peak means (green)and simCAS (purple).

Single-cell parameters were estimated for each cell type (Methods 5.2), then simulated and real datasets were compared using three core metrics: library size, peak mean, and cell sparsity. Distributional comparisons revealed that both simPIC(g) and simPIC(w) consistently mimicked the observed library size distributions across all datasets (Figure 2). In contrast, simCAS underestimated both the median and variance in several datasets, particularly PBMC5k, PBMC10k, Satpathy, and Liver. Similar trends were observed for peak mean distributions (Figure 2), where simCAS either overestimated (Cusanovich, Fly) or underestimated (PBMC5k, PBMC10k, Liver) the range of accessibility signals, while simPIC variants (gamma and weibull) more closely matched the real data. In the case of cell sparsity, simCAS generated matrices with substantially higher proportions of zero entries than observed in the real data (Figure 2), while simPIC more accurately preserved the sparsity profile characteristic of scATAC-seq data.

**Fig. 3:**
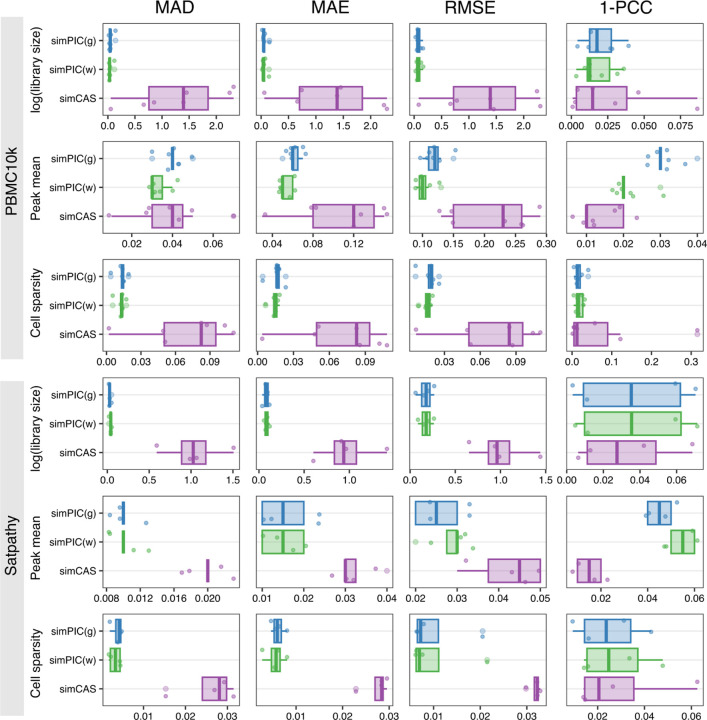
Comparative boxplots of key characteristics and scatter plots for relationships in PBMC5k(CD14 monocytes) dataset for simPIC(g) in blue, simPIC(w) in green and simCAS in purple. Error values are computed against the corresponding real dataset using four measures: the Median Absolute Deviation (the median of absolute differences, robust to outliers), the Mean Absolute Error (the average magnitude of absolute differences), the Root Mean Squared Error (the square root of the mean squared differences), and 1 minus the Pearson Correlation Coefficient (a dissimilarity metric where values closer to 0 indicate stronger correlation with the real data)

To quantify the fidelity of simulated data, we computed four distributional similarity metrics between real and simulated data: Mean Absolute Deviation (MAD), Mean Absolute Error (MAE), Root Mean Square Error (RMSE), and 1 - Pearson Correlation Coefficient (1 - PCC). We used these metrics to assess the performance of simulation methods with respect to library size, peak mean, and cell sparsity (Figure 3). simPIC consistently achieved lower error scores than simCAS across most datasets and features. For example, in the PBMC10k dataset, simPIC(w) achieved 96.6%, 13.6%, and 81.8% lower MAD values for log-library size, peak mean, and cell sparsity, respectively, compared to simCAS. While simCAS exhibited lower 1 - PCC values for peak means in certain datasets, suggesting stronger linear correlation with the real data, this came at the cost of larger absolute errors, particularly in distribution tails. In contrast, simPIC offered a better balance between trend preservation and distributional accuracy. Together, these results demonstrate that simPIC more faithfully reproduces the key statistical properties of real scATAC-seq data for individual cell types than simCAS.

#### 2.1.2 simPIC simulates multiple cell types and batch effects with configurable group structure

To assess simPIC’s ability to simulate heterogeneous samples, we extended our evaluation to include multiple cell types (referred to as groups) and technical batch effects, both of which are critical sources of variation in real scATAC-seq datasets. simPIC enables the user to flexibly specify the number of groups, their proportions, and the extent of differential accessibility between them. Additionally, batch effects—common technical artefacts introduced during sample collection, library preparation, or sequencing—can be incorporated through batch-specific multiplicative offsets on peak accessibility, thereby modeling variation orthogonal to biological structure.

##### Simulating multiple cell types with differential accessibility

We first evaluated group simulation using a subset of PBMC5k, PBMC10k data and Fly Brain data. In the two-group setting, we used nGroups = 2 and group.probs = c(0.5, 0.5) to generate two equally sized cell populations. The resulting simulated matrix captured distinct group-specific accessibility profiles, leading to two well-separated clusters in principal component analysis (PCA) space (Supplementary Figure S2 a–c), consistent with the intended 1:1 group ratio. To simulate more complex population structures, we next generated data with three groups and imbalanced sizes (nGroups = 3, group.probs = c(0.5, 0.25, 0.25)). As expected, the PCA projection (Supplementary Figure a-c, third column) showed three distinct clusters, with group sizes reflecting the specified proportions. These examples demonstrate that simPIC faithfully reproduces both biological heterogeneity and population structure, supporting its use in simulating realistic multi-cell-type scenarios.

##### Simulating batch effects across cell groups

To further model technical variation, we used simPIC’s batch simulation feature. Batch effects are introduced by applying multiplicative perturbations to peak accessibility values across subsets of cells, simulating technical variability independent of cell identity. In this setup, we generated two batches with equal distribution of cells from each group, specifying batch-level offsets while holding biological group structure constant (nGroups =2, group.probs =c(0,5,0.5), nBatches=2). The simulated data exhibited separation in lower-dimensional space not only by biological group but also by batch identity (Supplementary Figure S3), indicating that batch effects were successfully simulated.

### 2.2 simPIC is computationally efficient and scalable

To evaluate simPIC’s computational performance, we benchmarked CPU time and memory usage across varying simulation conditions (Methods 5.5). These metrics are critical for scalable single-cell simulations, particularly when generating large datasets or conducting parameter sweeps.

**Fig. 4:**
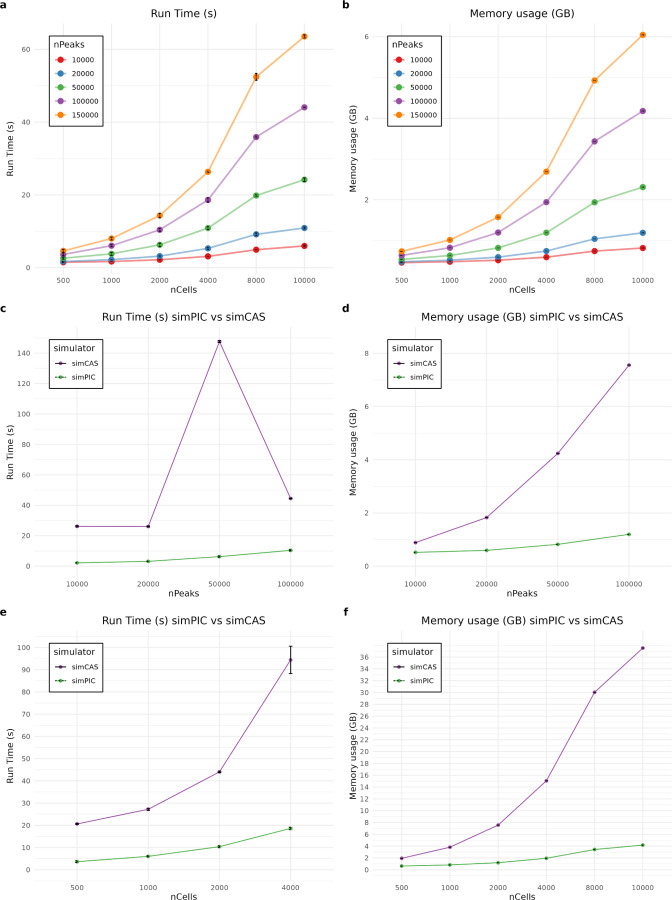
Comparison of computational performance of simPIC and simCAS over five simulations. (a) simPIC processing time varying nCells and nPeaks. (b) simPIC memory usage varying nCells and nPeaks. (c) Processing time comparison between simPIC and simCAS varying nPeaks and keeping nCells as 2,000. (d) Memory usage comparison between simPIC and simCAS varying nPeaks and keeping nCells as 2000. (e)

We first assessed how simPIC scales with increasing dataset size. Simulations were performed with varying numbers of peaks and cells using the default simPIC(w) mode. As expected, both runtime and memory usage increased smoothly with larger matrix dimensions (Figure 4a,b). Notably, simPIC was able to generate a count matrix with 100,000 peaks and 10,000 cells in approximately 44 seconds using 4.2 GB of memory, demonstrating its compatibility with standard computing environments, including personal laptops.

We then compared simPIC’s performance with simCAS with pseudo-cell-type modeling. In one benchmark, we fixed the number of cells to 2,000 and varied the number of peaks from 10,000 to 100,000. simPIC consistently outperformed simCAS in both speed and memory usage (Figure 4c,d). For example, simPIC simulated a dataset with 10,000 peaks and 2,000 cells in 2 seconds and 0.5 GB RAM, whereas simCAS required 26 seconds and 0.88 GB for the same task. This performance gap widened with increasing peak number: simPIC completed the 100,000 peak simulation in 10.4 seconds using 1.1 GB, while simCAS required 44.5 seconds and 7.6 GB. Interestingly, simCAS exhibited a non-monotonic trend in runtime as peak numbers increased requiring less time for 100,000 peaks than for 50,000 peaks—likely due to stochastic variation in internal sampling or memory management. In contrast, simPIC showed consistent scaling behavior. In a complementary benchmark, we fixed the number of peaks at 100,000 and varied the number of cells (Figure 4e,f). Again, simPIC demonstrated superior performance, maintaining low runtime and memory usage even for large cell numbers. At 10,000 cells, simPIC completed the simulation in 44 seconds and 4.2 GB RAM, whereas simCAS required 1,090 seconds and 37.5 GB, highlighting substantial computational overhead.

### 2.3 Simulating population scale data with genetic effects

To evaluate simPIC’s ability to simulate biologically realistic inter-individual variation in chromatin accessibility, we applied it to generate population-scale scATAC-seq data incorporating genetic effects. We benchmarked these simulations against a publicly available Alzheimer’s disease dataset [18], at the time of writing the only open-access scATAC-seq resource that includes both genotype and cell-type-resolved accessibility data across individuals. Following an approach similar to splatPop [5], we estimated cell-level parameters from the individual with the most high-quality cells and derived population-level parameters from pseudo-bulk aggregated profiles. Using these estimates, we simulated accessibility counts for one cell type across multiple individuals and compared the structure of real and simulated data in lower-dimensional space.

**Fig. 5:**
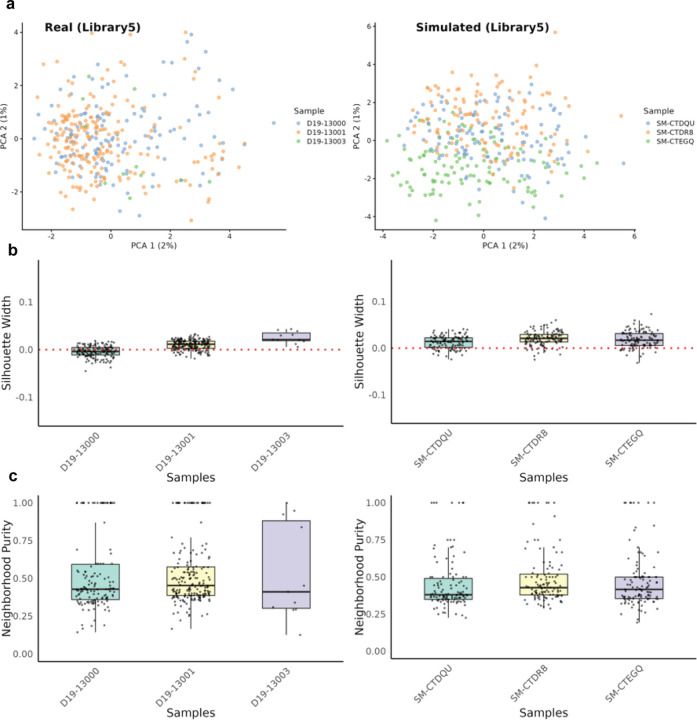
Quantifying clustering in real and simPIC-simulated data with inter-individual genetic variation. (a) PCA plot of real and simulated data, coloured by individual, showing the preservation of individual specific structure. (b) Silhouette width comparison between real and simulated data, assessing cluster compactness. (c) Neighbourhood purity analysis, evaluating local structure consistency across real and simulated datasets.

In both real and simulated datasets, cells tended to cluster by individual in PCA space, with similar degrees of overlap and 2% variance explained by the top two components (Figure 5). A distinct cluster in the real data, corresponding to one donor (D19–13182), was absent in the simulation, likely due to unmodeled technical variation. Nonetheless, the global structure was preserved, indicating that simPIC effectively captures biological variability across donors.

We further assessed simPIC’s performance using the microglia population (library 5) from the same dataset, comprising 344 cells from three individuals and 1,693 peaks. Both real and simulated data showed comparable global structure and variance explained by principal components (Figure 5a). Silhouette scores, used to assess clustering by donor, were similarly distributed between datasets, as were neighborhood purity scores (Figure 5b,c) calculated using the bluster R package [19], suggesting that local and global individual-specific patterns are realistically captured. Comparable results were obtained across additional libraries from early-stage and control individuals, confirming the robustness of simPIC’s population-scale simulation framework(Supplementary Figures S4–S10).

**Fig. 6:**
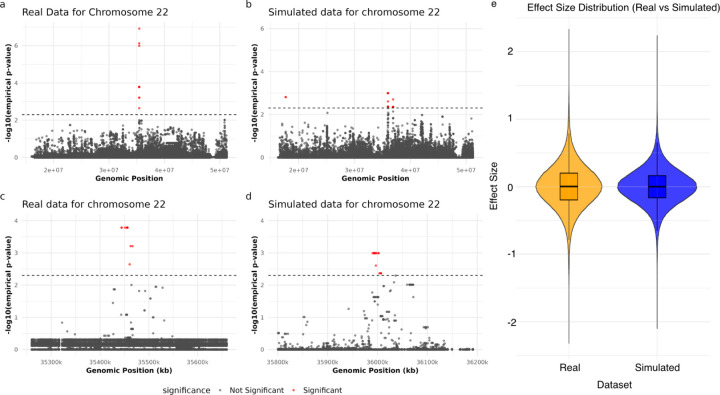
Comparison of QTL mapping results between real and simulated data for chromosome 22. (a-b) Manhattan plots showing −log10(empirical p-values) of SNP associations in (a) real data and (b) simulated data, with horizontal dashed line indicating significance threshold < 0.005. (c-d) Zoomed-in views of 400kb regions showing similar clustering patterns of significant variants despite positional differences. Right panel shows Violin and box plots comparing effect size distributions between real and simulated datasets, demonstrating remarkably similar statistical properties despite differences in specific significant SNP positions.

To assess simPIC’s ability to model genetic effects, we performed cis-caQTL mapping using LIMIX [20] on both real and simulated microglia data for chromosome 22. We tested all SNP–peak pairs within a ±100 kb window using a linear mixed model that accounts for relatedness and technical covariates. In the real dataset, we identified 14 significant associations at an empirical p-value threshold of < 0.005; the corresponding simulated dataset yielded 46 associations under the same threshold (Figure 6a). While no individual SNPs overlapped between the datasets, the overall number of discoveries was similar, suggesting comparable statistical power and a realistic representation of inter-individual variability in the simulation. To investigate the distribution of associations in genomic context, we examined two 400kb genomic windows with high association density in each dataset. In both cases, associated SNPs were spatially clustered within these regions (Figure 6a–d), demonstrating that simPIC preserves local enrichment patterns driven by linkage disequilibrium typical of regulatory hotspots, even without reproducing exact loci. Finally, we compared the distributions of effect sizes across significant SNP–peak pairs. Both datasets exhibited nearly identical effect-size distributions (Figure 6e), suggesting that simPIC accurately reproduces the genome-wide spectrum of genetic effect magnitudes. The lack of overlap in specific SNPs is expected, as simPIC samples genotypes and assigns peak-specific effect sizes probabilistically, without using real haplotypes or linkage disequilibrium (LD) structure. Nevertheless, the simulation captures key statistical properties of QTL landscapes, including effect-size distributions and regional aggregation of associations. Together, these results demonstrate that simPIC can simulate population-scale single-cell chromatin accessibility datasets with genetic effects that closely mirror real data in structure, variability, and association statistics, making it a valuable tool for benchmarking QTL discovery pipelines and modelling regulatory variation across individuals.

## 3 Discussion

The shift of single-cell technologies such as scATAC-seq to population-scale studies has created unprecedented opportunities for dissecting the genetic regulation of chromatin accessibility, but it has also introduced new analytical challenges. Reliable simulation tools are essential to develop and evaluate methods capable of handling the complexity of these datasets. The R/Bioconductor package simPIC provides a flexible and interpretable framework for simulating scATAC-seq data that incorporates key features of real experiments. By estimating parameters from empirical data and allowing fine-tuning through user-defined controls, simPIC balances realism with usability. Its integration with splatPop extends its utility to studies of population structure and caQTLs, while reproducibility and usability are ensured through extensive documentation and code availability.

A defining strength of simPIC is its ability to model genetic effects at population scale, making it uniquely suited for benchmarking caQTL mapping approaches. By simulating ground-truth causal variants, effect-size distributions, and realistic linkage disequilibrium (LD) patterns, simPIC allows systematic evaluation of association methods under different experimental conditions. Researchers can assess statistical power across cohort sizes, test caQTL mapping accuracy in high-LD regions, and examine the reproducibility of QTL detection in the presence of batch effects or cell-type-specific heterogeneity. This functionality directly addresses a critical constraint on method development for caQTL mapping, namely the limited availability of real caQTL datasets, which remain costly and scarce.

By explicitly modelling batch effects and cell-group variability, simPIC provides a platform for systematic evaluation of preprocessing and downstream analysis steps. Users can generate datasets with tunable levels of sparsity, sequencing depth, and technical variation to benchmark normalization and batch-correction procedures. Likewise, controlled heterogeneity across cell groups enables targeted assessments of clustering, annotation, and feature selection methods, ensuring that observed performance reflects recovery of the true underlying accessibility structure rather than artefacts of the data.

The utility of simPIC also extends to emerging computational approaches. By providing datasets with a known underlying structure, specifically effects of individual variants on chromatin accessibility, simPIC could plausibly be used to assess methods for predicting variant effects. In addition, the ability to simulate multi-individual datasets enables preliminary benchmarking of integrative analyses that combine chromatin accessibility with other modalities, where real ground truth is rarely available. These applications highlight how simPIC can complement existing benchmarking resources without replacing the need for empirical validation.

Like all simulators, simPIC has limitations. Exact positional concordance of significant SNPs with those observed in empirical datasets is unlikely, reflecting simplified assumptions about recombination and effect-size distributions. Nonetheless, simPIC successfully captures higher-order structural properties that represent core features of complex trait architecture. Current gaps include the lack of conditional simulations across disease states, time points, and the absence of explicit modelling of epigenetic modifications or chromatin interactions. Future extensions that incorporate these aspects, as well as multi-omic layers linking accessibility to gene expression or proteomics will further expand its scope and impact.

In summary, simPIC provides a realistic, flexible, and reproducible framework for simulating scATAC-seq data that incorporates genetic effects, batch structure, and cell group variability. By complementing scarce population-scale datasets, simPIC enables rigorous benchmarking of analytical pipelines ranging from caQTL mapping to clustering, and computational modelling. Its modular design ensures adaptability to evolving study designs, positioning simPIC as a valuable foundation for advancing reproducible and scalable single-cell epigenomics research.

## 4 Conclusion

Here, we introduced simPIC, a flexible and extensible framework for simulating population-scale single-cell chromatin accessibility data. Implemented in R and available via Bioconductor under a GPL-3 license, simPIC builds on real genotype data to simulate genetic effects on chromatin accessibility. This software enables simulation of realistic population structure and supports caQTL mapping applications. In addition to modelling genetic effects, simPIC incorporates technical variability such as batch effects and biological variability such as cell group-specific accessibility patterns. Together, these features allow users to create synthetic datasets that reflect the complexity of real scATAC-seq experiments.

## 5 Methods

### 5.1 Datasets and pre-processing

All scATAC-seq datasets used in this study were processed uniformly to reduce variation arising from different pre-processing approaches and sequencing technologies (Table 1). Datasets were first downloaded from the Gene Expression Omnibus (GEO) [21]. Peak-by-cell count matrices were generated using the PIC_counting function from the PICsnATAC R package (v0.2.3) [22]. The resulting matrices were then imported into ChromatinAssays using the Signac package (v1.11.9) [23] and Seurat (v4.9.9) [24] without any additional filtering (min.cells=min.features= 0). Cell type labels were assigned using existing metadata when available, or by label transfer from paired single-cell RNA sequencing data for the 10x Genomics datasets. Peaks were filtered to include only those detected in at least 1% of cells within each cell type and dataset. Cell types with fewer than 200 cells were excluded to ensure sufficient data for analysis. Finally, the filtered datasets were converted into SingleCellExperiment (SCE) objects for downstream processing. The datasets used in this study include:

- 10x Genomics: PBMC10k (ID:10k_pbmc_ATACv2_nextgem_Chromium_Controller) and PBMC5k (ID:atac_v1_pbmc5k) dataset.
- Satpathy dataset [14]: processed count matrix, fragment files and metadata files downloaded from GEO (GSE129785).
- Fly brain dataset [17]: peak regions, cell barcodes, and cell metadata were extracted from the cisTopic object L3P12_cisTopic.Rds, downloaded from flybrain. We excluded cell types labeled as unknown (CellType_lvl1 equal to ‘unk’ or ‘-’).
- Cusanovich dataset [16]: BAM files and cell metadata were downloaded from mouse-atac. Fragment files required as input for PIC_counting were generated using Python package Sinto (v0.10) sinto with default parameters. This dataset consists of chromatin accessibility profiles across 13 different mouse tissues, from which we randomly selected three tissues: cerebellum, kidney, and spleen.
- Liver dataset: fragment files were downloaded from GEO (GSE199799) and processed using ArchR as detailed in [15].

For the population-scale study the Alzheimer’s disease dataset was used from Xiong et.al [18], downloaded from Synapse portal as described in the original paper. The dataset was pre-processed to match sample names with individual IDs resulting in a total of 83 individuals.

### 5.2 simPIC simulation framework

The simPIC simulation framework has four key components: (i) estimating parameters from empirical data at both single sample and population levels, (ii) simulating cell groups and batches for a single sample, (iii) modeling peak means with genetic effects across a population, and (iv) simulating single-cell counts for individual and population-scale data.

#### Parameter estimation

##### a) Single-sample parameter estimation

To generate realistic scATAC-seq data from a single sample, simPIC estimates three key parameters from a user input peak-by-cell count matrix or SingleCellExperiment (SCE) object using the simPICestimate function: library size, peak-wise mean accessibility, and cell-level sparsity. Library size is modeled using a log-normal distribution, with parameters mean $\left(\mu \right)$ and standard deviation $\left(\sigma \right)$. Peak-wise mean accessibility is computed after normalizing each cell to the dataset’s median total count. Peaks with zero counts across all cells are removed, and a Weibull distribution is fit to the non-zero peak means using the fitdistrplus package [25] to estimate shape $\left(\eta \right)$ and scale $\left(\kappa \right)$ parameters. simPIC also provides an array of alternative distributions from the generalized gamma family (e.g., gamma, Pareto, lognormal-gamma mixture) to model peak-wise mean accessibility which can be used if they better fit the data (see Supplementary Note 1). Cell-level sparsity is modeled by estimating the proportion of zero entries per cell and fitting a Bernoulli distribution with parameter π.

When simulating multiple cell groups or subpopulations (nGroups > 1) within a single sample, parameter estimation is performed separately within each group, allowing group-specific variation in accessibility and sparsity profiles. When batch effects are included (nBatches > 1), they are incorporated either by applying multiplicative factors to each peak across all cells within a batch or by using user-defined batch labels. In cases where both multiple groups and batches are specified, simPIC models biological variability using the biological coefficient of variation (BCV), which captures the mean–variance relationship typical of scATAC-seq data [26, 27]. BCV is modeled using a scaled inverse chi-squared distribution, with parameters ϕ and $df_{0}$ estimated via the estimateDisp function in edgeR [28]. Since edgeR tends to overestimate the common dispersion in synthetic data [4], we applied a linear correction to the estimate to better match observed variance patterns: $\hat{\phi }\;=\;-0.3+0.15{\hat{\phi }}_{\text{edgeR}}$ (Supplementary Figure S1). All estimated parameters are stored in a simPICcount object for downstream simulation. Default settings are available for exploratory runs, and all parameters can be customized by the user.

##### b) Population-scale parameter estimation

To simulate population-scale single-cell ATAC-seq data, we extended simPIC by incorporating inter-individual variability using an approach adapted from splatPop [5]. Starting with a peak-by-cell count matrix annotated with individual identities, accessibility counts were aggregated across cells to generate a peak-by-individual matrix for parameter estimation. A two-step procedure was applied to capture both global accessibility trends and individual-level heterogeneity. First, for each sample j, peak-mean accessibility $\left(\eta_{j}\right)$ and dispersion $\left(\kappa_{j}\right)$ were estimated by fitting a Weibull distribution to the empirical distribution of mean accessibility values across individuals. Second, to account for the mean–variance relationship, peaks were grouped into bins of 50 based on mean accessibility (as in splatPop). The coefficient of variation (CV) was computed within each bin, and a gamma distribution was fit to these values to estimate bin-specific shape $\left(\alpha_{v}\right)$ and rate $\left(\beta_{v}\right)$ parameters. Parameters controlling for caQTL effect sizes $\left(\alpha_{c}\right)$ and $\left(\beta_{c}\right)$ were estimated by fitting a gamma distribution to effect sizes from an empirical caQTL mapping study [18]

###### Simulating cell-groups and batches for single sample

simPIC simulates samples with multiple cell groups by defining distinct cell groups where peaks exhibit differential accessibility. Consistent with previous reports indicating that approximately 13% of peaks are differentially accessible in single-cell ATAC-seq data [29], a multiplicative differential accessibility (DA) factor is assigned to each peak i and applied to its baseline mean $\left(\lambda_{i}\right)$. DA factors for differentially accessible peaks are sampled from a log-normal distribution, while non-differential peaks have a DA factor set to one. The number of groups and cell membership probabilities are user-defined. Additionally, parameters governing the probability of DA, and the magnitude and direction of DA factors, can be specified for each group. The resulting single-cell experiment object contains group assignments and corresponding DA factors for each cell.

Batch effects represent technical variation introduced during sample handling, preparation, or processing that can obscure true biological signals. simPIC models these effects by applying multiplicative scaling factors to peak accessibility values across cells within batches. For each cell j in batch b, the scaling factor $\omega_{j}^{b}$ is drawn from a log-normal distribution, $\omega_{j}^{b}LN\left(\mu_{b},\;\sigma_{b}\right)$, where $\mu_{b}$ and $\sigma_{b}$ control the magnitude and variability of the batch effect. Users specify the number of batches *nBatches* and the number of cells per batch *batchCells*, with the total number of cells equal to the sum of *batchCells*. Adjusting these parameters allows control over the degree of batch variation, with larger or more variable scaling factors producing stronger batch effects.

###### Modelling peak means with genetic effects across a population

To simulate population-scale single-cell ATAC-seq data, we created a wrapper around splatPop extending the simPIC framework [5] by adapting splatPop’s gene-based model to chromatin accessibility peaks. For each peak i, population-wide accessibility means $\left(\lambda_{i}\right)$ were sampled from a gamma distribution with parameters $\eta_{j}$ and $\kappa_{j}$. Peak-specific variability $\left(\sigma_{i}\right)$ was also sampled from a gamma distribution, with an optional scaling factor $\left(s\right)$ to adjust overall variability. Baseline accessibility for each peak in each individual $\left(\lambda_{i,j}\right)$ was drawn from a normal distribution centered on $\left(\lambda_{i}\right)$ and scaled by $\left(\sigma_{i}\right)$. When empirical data were provided to the splatPopSimulate function, these sampling steps were bypassed and empirical means and variances were used directly.

To simulate caQTL effects, we used real genotype and peak annotation data from [18], including VCF and GFF files. Parameters such as the proportion of accessible peaks associated with caQTLs (caPeaks), the minor allele frequency of the associated SNPs (caSNPs), and the distance between each caPeak and its linked SNP were defined to reflect the population structure. caQTL effect sizes $\left(\omega_{i}\right)$ were sampled from a gamma distribution with parameters $\alpha_{c}$ and $\beta_{c}$ and applied to peak accessibility based on genotype ($G_{i,j}$, encoded as 0, 1, or 2). The framework also supports group-specific caQTLs that affect only certain cell groups, and condition-specific effects tailored to specific cohorts.

Differential accessibility (DA) between groups or conditions was simulated by scaling peak accessibility using factors drawn from a log-normal distribution with parameters $\left(\mu_{\text{da}},\;\sigma_{\text{da}}\right)$. Separate parameters were used for cell groups and cohorts to control these effects independently. The model also allowed specification of the proportion of peaks with reduced accessibility (negative DA effects), enabling flexible control over heterogeneity across the simulated dataset.

###### Simulating single-cell counts for individual and population scale data

To simulate a scATAC-seq peak-by-cell count matrix, we used two core functions in the simPIC framework: simPICsimulate for one cell group and simPICsimulateGroup for multi-group or batch simulations. In both cases, peak accessibility means $\left(\lambda_{i}\right)$ are drawn from a Weibull distribution and cell-specific library sizes $\left(L_{j}\right)$ are sampled from a log-normal distribution. When simulating one cell group, simulated library sizes are used to generate the counts matrix $Y_{ij}$ by sampling from a Poisson distribution with mean parameter $\left({\bar{\lambda }}_{i}L_{j}\right)$ and sparsity is introduced by a sparsity indicator $Z_{ij}$ controlled by parameter $\pi_{j}$ which randomly zeros out entries using a Bernoulli-distribution to reflect highly sparse nature of scATAC-seq data. When simulating multiple cell groups and batches, peak means are normalized and scaled by library size to compute adjusted values $\left(\lambda_{i,j}\right)$. Cell-specific accessibility means $\left(\lambda_{i,j}\right)$ are then sampled from a gamma distribution using these adjusted means. Biological variability is further captured using a biological coefficient of variation (BCV), computed from user-defined parameters $\left(\phi ,\;df_{0}\right)$ and used to scale dispersion across peaks. Larger mean values result in lower BCV, modeling the inverse relationship between accessibility and variability. Counts are then sampled and sparsity introduced as in simPICsimulate. Population scale data is simulated as described in splatPop.

### 5.3 Comparative analysis of simulation frameworks

We used cell-type-specific preprocessed SCE objects to generate synthetic scATAC-seq data using simPIC. For benchmarking, We compared simPIC with simCAS, but did not include simATAC [7], as it simulates pseudobulk data [8] and outputs a bin-by-cell matrix, which is not directly comparable. To run simCAS, we converted the SCE object to AnnData format (as required by simCAS) using the SCE2AnnData function from the zellkonverter R package (v3.18) [30]. Simulations were performed in pseudo-cell-type mode using the command-line interface, which aligns with simPIC’s setup for consistent comparison. The simulated output was converted back to SCE format using AnnData2SCE. Both simPIC and simCAS were used to simulate the same number of cells and peaks as in the real dataset. This comparison was limited to a single cell type and did not include multi-group or multi-batch settings. Furthermore, since no existing tool simulates population-scale scATAC-seq data with inter-individual genetic variation, we did not perform comparisons in that setting. Simulated data were evaluated against real data using the simPICcompare function, which generates diagnostic plots to assess similarity across key features.

### 5.4 Evaluation metrics

We evaluated the simulation performance of simPIC (with Weibull, simPIC(w), and gamma, simPIC(g) distributions) and simCAS, focusing on three key characteristics: library size, peak mean, and cell sparsity. To assess similarity with real data, we computed four metrics: Median Absolute Deviation (MAD), Mean Absolute Error (MAE), Root Mean Square Error (RMSE), and 1 minus Pearson Correlation Coefficient (1−PCC). These were calculated from the absolute differences between sorted real and simulated values for each characteristic. We also compared the distributions of six feature-level and two cell-level summary statistics: peak means, peak variance, proportion of zeros per peak, the relationship between peak mean and percent zeros, the correlation between peak mean and peak variance, and the relationship between peak mean and non-zero proportion; along with library size and cell sparsity at the cell level. Together, these metrics provide a comprehensive assessment of how well the simulations recapitulate real data characteristics.

### 5.5 Computational efficiency

To assess the computational efficiency of simPIC and simCAS, we measured CPU time and memory usage across simulated datasets with varying numbers of cells (500, 1,000, 2,000, 4,000, 8,000, and 10,000) and peaks (10,000, 20,000, 50,000, 100,000, and 150,000), while keeping other parameters at default values. Each configuration was run five times. CPU time and memory usage were measured from the point of reading the input real data to the completion of simulated peak-by-cell matrix generation. For simPIC, elapsed time was recorded using R’s system.time, and peak memory was measured using the mem_used function from the pryr package (v0.1.6). For simCAS, both CPU time and memory usage were recorded using Python’s time and tracemalloc modules, respectively.

### 5.6 caQTL mapping analyses

We simulated a larger dataset based on parameter estimates from library 11 of the microglia cell type from the Xiong et.al, dataset [18]. The simulation included 100 cells per individual for 83 samples, matching the real dataset, within a single batch. caQTL effects were assigned to 70% of the simulated peaks. Single-cell counts were normalized using scran (v1.28.2), then aggregated by individual through mean aggregation and quantile normalized to a standard normal distribution [31]. caQTL mapping was conducted on all SNPs within 100 kb upstream and downstream of each peak on chromosome 22 using a linear mixed model (LMM) implemented in LIMIX [20]. The model included SNP genotype as a fixed effect, the top 15 principal components from chromatin accessibility PCA as fixed effects to control unwanted variation, and a kinship matrix calculated with PLINK (v1.90) [32] as a random effect to adjust for population structure. Associations with empirical feature p-values below 0.005 were considered significant. Manhattan plots showing the full chromosome 22 and regional 400 kb windows were generated for both real and simulated data.

## Supplementary Material

Supplement 1

## Figures and Tables

**Table 1:** Datasets used in this study

Dataset:Year	Organism	Platform	Sequencing	Accession No
PBMC5k:2021	*Homo sapiens*	10x Chromium-(snATAC)	Illumina Novaseq 6000	NA
PBMC10k:2022	*Homo sapiens*	10x Chromium-(snATAC)	Illumina Novaseq 6000	NA
Sathpathy:2019	*Homo sapiens*	10x Chromium-(scATAC)	Illumina HiSeq 2000	GEO: GSE129785
Buquicchio:2022	*Mus musculus*	10x Chromium-(scATAC)	Illumina HiSeq 4000	GEO: GSE199799
Cusanovich:2018	*Mus musculus*	sciATAC	Illumina HiSeq 2500	GEO: GSE111586
Fly Brain:2022	*Drosophila melanogaster*	scATAC	NextSeq500 and NovaSeq6000	GEO: GSE163697

## Data Availability

simPIC is available on Bioconductor https://bioconductor.org/packages/release/bioc/html/simPIC.html and on GitHub https://github.com/sagrikachugh/simPIC. Data and code to reproduce the figures in this manuscript are available under CC BY license on GitHub repository https://github.com/sagrikachugh/simPIC-paper, a website accompanying the analysis is available here https://sagrikachugh.github.io/simPIC-paper/
