# Supplementary material for: simPIC:flexible simulation of paired-insertion counts for single-cell ATAC sequencing data
Source: bioRxiv. 2025 Sep 23:2025.09.21.676689. Preprint. [Version 1] doi: 10.1101/2025.09.21.676689 (PMC12485805; doi:10.1101/2025.09.21.676689)
Supplement: Supplement 1 [file media-1.pdf]

Supplementary information for simPIC

Sagrika Chugh[1,2], Heejung Shim[2], Davis J. McCarthy[1,2,3*]

[1]Bioinformatics and Cellular Genomics, St. Vincent's Institute of Medical Research, 9 Princes Street, Fitzroy, 3065, Victoria, Australia.
[2]School of Mathematics and Statistics, Faculty of Science, University of Melbourne, Grattan Street, Parkville, 3010, Victoria, Australia.
[3]Faculty of Medicine, Dentistry and Health Sciences, University of Melbourne, Grattan Street, Parkville, 3010, Victoria, Australia.

*Corresponding author(s). E-mail(s): dmccarthy@svi.edu.au;
Contributing authors: schugh@svi.edu.au;
heejung.shim@unimelb.edu.au;

Table S1: No of peaks after 1% filter

Dataset	Celltype	nCells	nPeaks	nPeaks (1%filter)
PBMC5k	CD14 Monocytes	1768	69523	56279
	CD4 Naive	432	69523	45140
	CD8 Effector	505	69523	51493
	Double negative T cell	286	69523	46534
	CD4 Memory	641	69523	47538
	CD8 Naive	278	69523	43410
PBMC10k	CD14 Monocytes	2201	127689	106967
	CD4 Memory	2419	127689	83919
	CD4 Naive	1033	127689	78962
	CD8 Effector	866	127689	81813
	B cell progenitor	203	165434	97785
	CD8 naïve	440	165434	84291
	NK dim	760	165434	91594
Satpathy	CD 34 Progenitors	14633	571400	175151
	Monocytes	1868	571400	144533
	CD8 Naive	1945	571400	83386

Continued on next page...

Dataset	Celltype	nCells	nPeaks	nPeaks (1%filter)
	Regulatory T cells	2598	571400	92542
Buquicchio	Liver TRM	4618	67364	64428
Fly Brain	OL Type I NB	323	51135	42926
	OL Neuroepithelium	274	51135	49296
	Capa	216	51135	42196
	CB Type I NB	205	51135	43759
Cusanovich (Spleen)	B cells	2197	73177	62219
Cusanovich (Cerebellum)	Cerebellar granule cells	1091	67260	37376
	Astrocytes	363	67260	36496
Cusanovich (Kidney)	Podocyte	447	101900	40426
	Proximal Tubule	2565	101900	85796

Dispersion estimate

It is well established that scATAC-seq data exhibits a strong mean-variance trend, where regions with higher mean accessibility tend to show greater variability [1, 2]. This pattern arises due to inherent biological variability, technical noise in single-cell measurements and fundamental statistical property of count data. To accurately replicate this trend in simulated data, I enforce it in extended simPIC framework (Main Figure 1) by estimating biological coefficient of variation (BCV) from real data, with parameters ϕ and df_0 from a scaled inverse chi-squared distribution using the `estimateDisp` function from the `edgeR` package [3], where the scaling factor is a function of the peak mean. This ensures that simulated datasets capture realistic variability patterns observed in real single-cell ATAC-seq experiments. However, upon testing this estimation procedure on simulated datasets, I observed that the `edgeR` estimate of common dispersion was inflated, as shown in Figure S1. This inflation has also been reported in the simulation of single-cell RNA-seq data using the `splatter` package [4]. To address this issue, I applied a linear correction to the edgeR-estimated dispersions using the formula $\hat{\phi} = -0.3 + 0.15\hat{\phi}_{\texttt{edgeR}}$. This correction helps to adjust for the overestimation of dispersion, leading to more accurate simulated data that better reflects the true variability observed in real datasets.

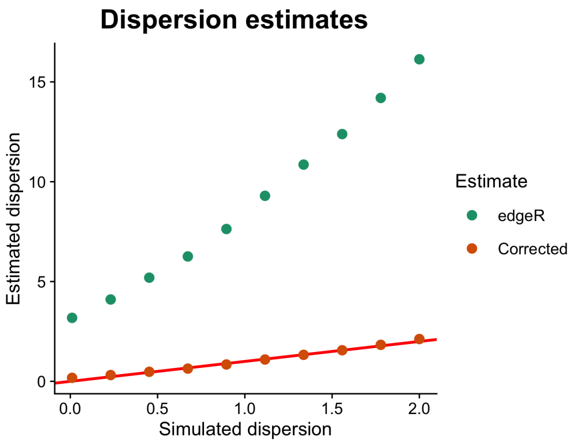

Fig. S1 Scatter plot of estimated dispersions against the true simulated values. Estimates of common dispersion obtained from edgeR (green) can be inflated for single-cell data. The simPIC simulation uses a linearly corrected value (orange) in its estimation procedure. The red line shows the true values estimates.

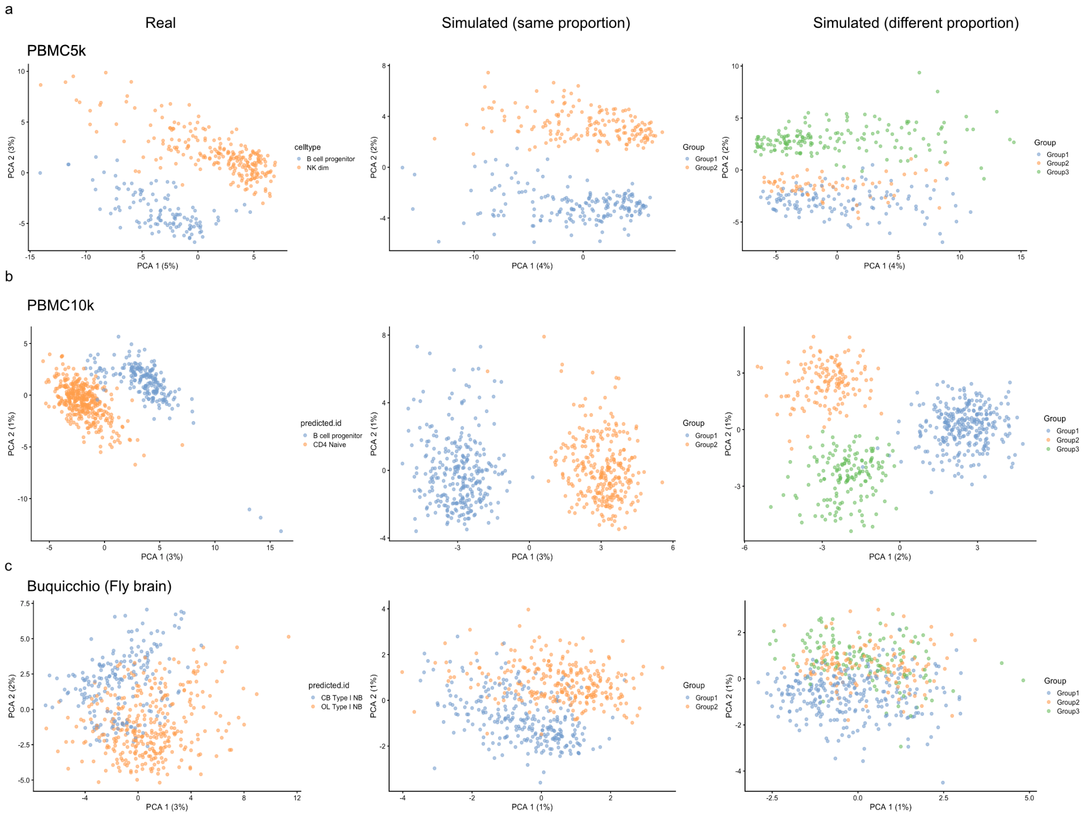

Fig. S2 Principal Component Analysis (PCA) plot illustrating the separation of two simulated cell type groups generated using simPIC. Group 1 is represented in blue, and Group 2 in orange, demonstrating that simPIC can simulate multiple cell types while maintaining the same or different proportions across groups.

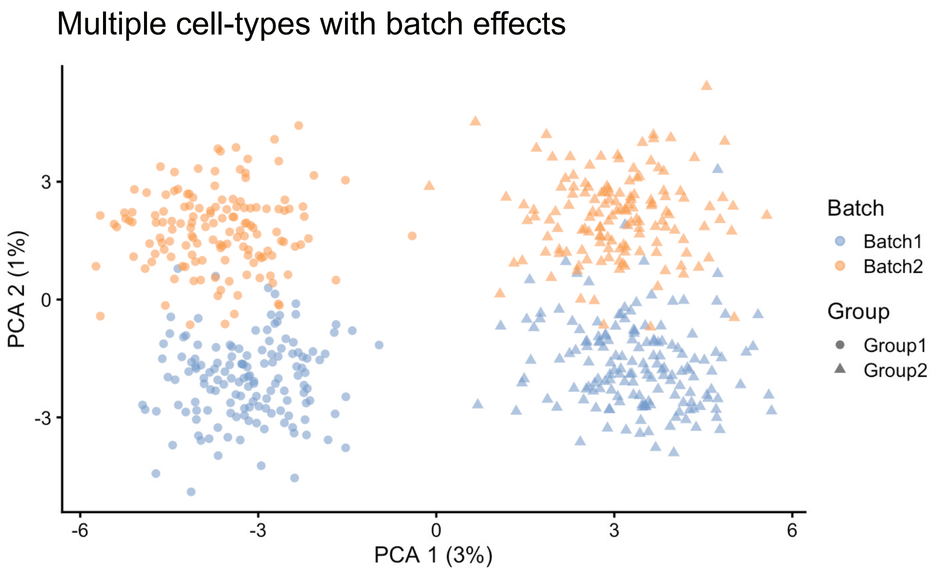

Fig. S3 Principal Component Analysis (PCA) plot showing two simulated groups and batches generated using simPIC. Group 1 is represented by circles, and Group 2 by triangles, while Batch 1 is coloured in blue and Batch 2 in orange, demonstrating the ability of simPIC to simulate batch effects across groups.

Clustering diagnostics

Clustering diagnostics were done for multiple libraries across the Alzheimer's disease (AD) and non Alzheimer's category. The following figures show all the different examples with varying no of cells for each library

The below figures are for earlyAD samples

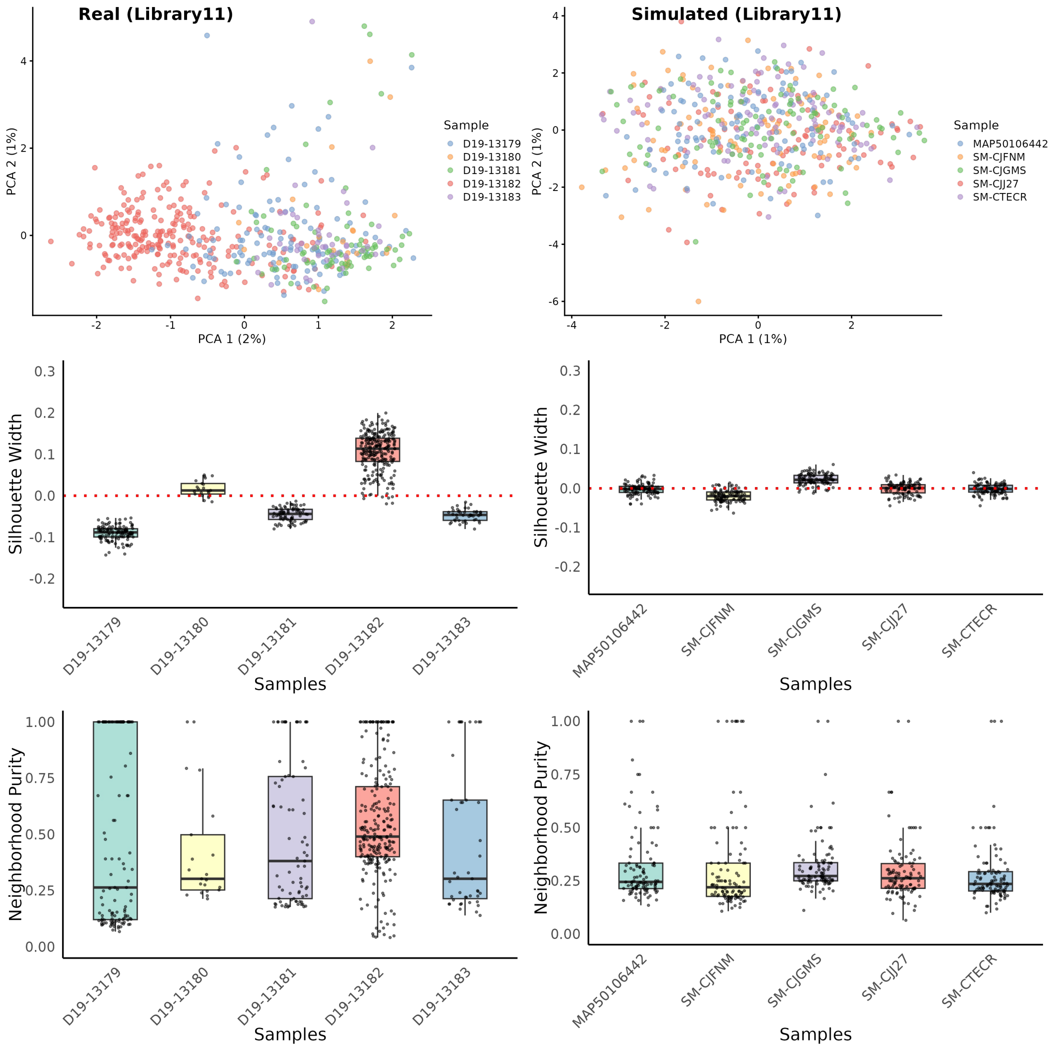

Fig. S4 Quantifying clustering in real and simPIC-simulated data for early AD library 11 with 1693 peaks and 507 cells. (Top row) PCA plot of real and simulated data, coloured by individual, showing the preservation of individual-specific structure. (Middle row) Silhouette width comparison between real and simulated data, assessing cluster compactness. (Bottom row) Neighbourhood purity analysis, evaluating local structure consistency across real and simulated datasets.

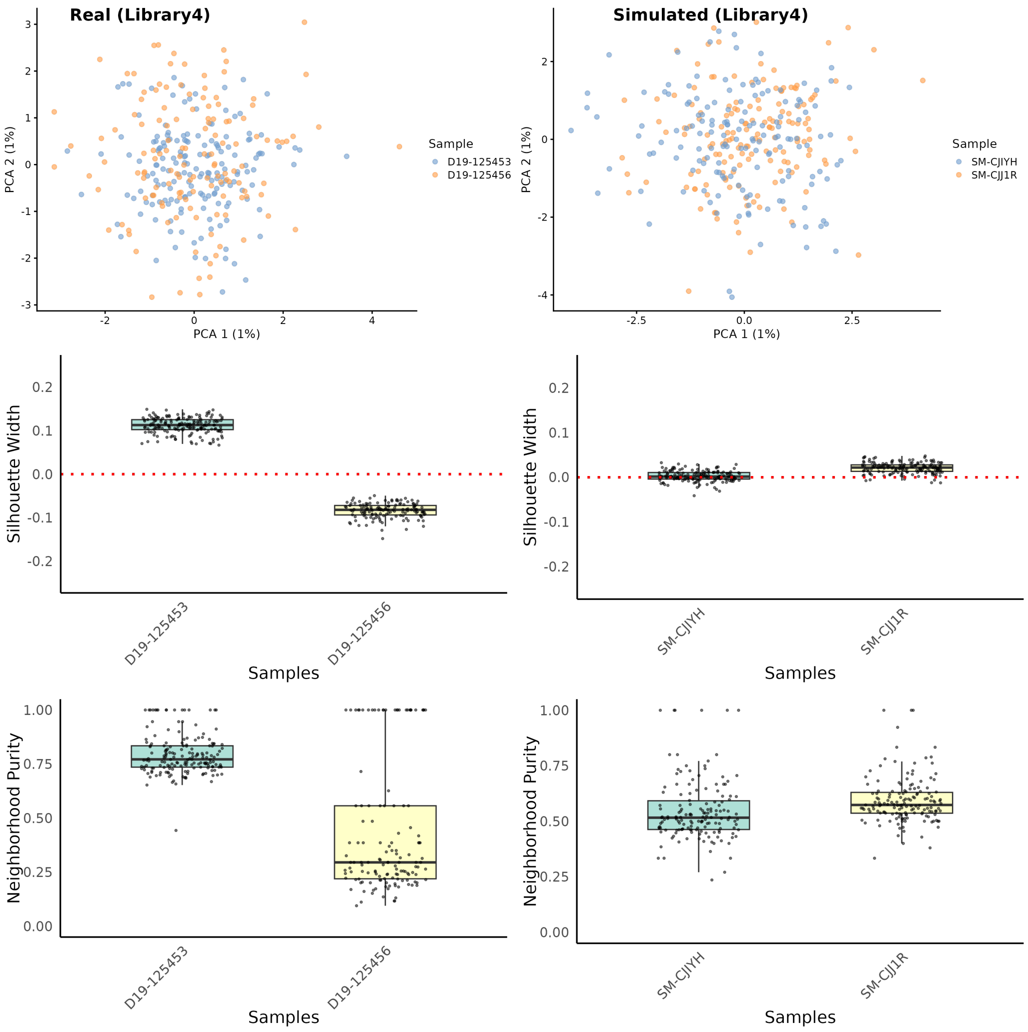

Fig. S5 Quantifying clustering in real and simPIC-simulated data for early AD library 4 with 1693 peaks and 307 cells. (Top row) PCA plot of real and simulated data, coloured by individual, showing the preservation of individual-specific structure. (Middle row) Silhouette width comparison between real and simulated data, assessing cluster compactness. (Bottom row) Neighbourhood purity analysis, evaluating local structure consistency across real and simulated datasets.

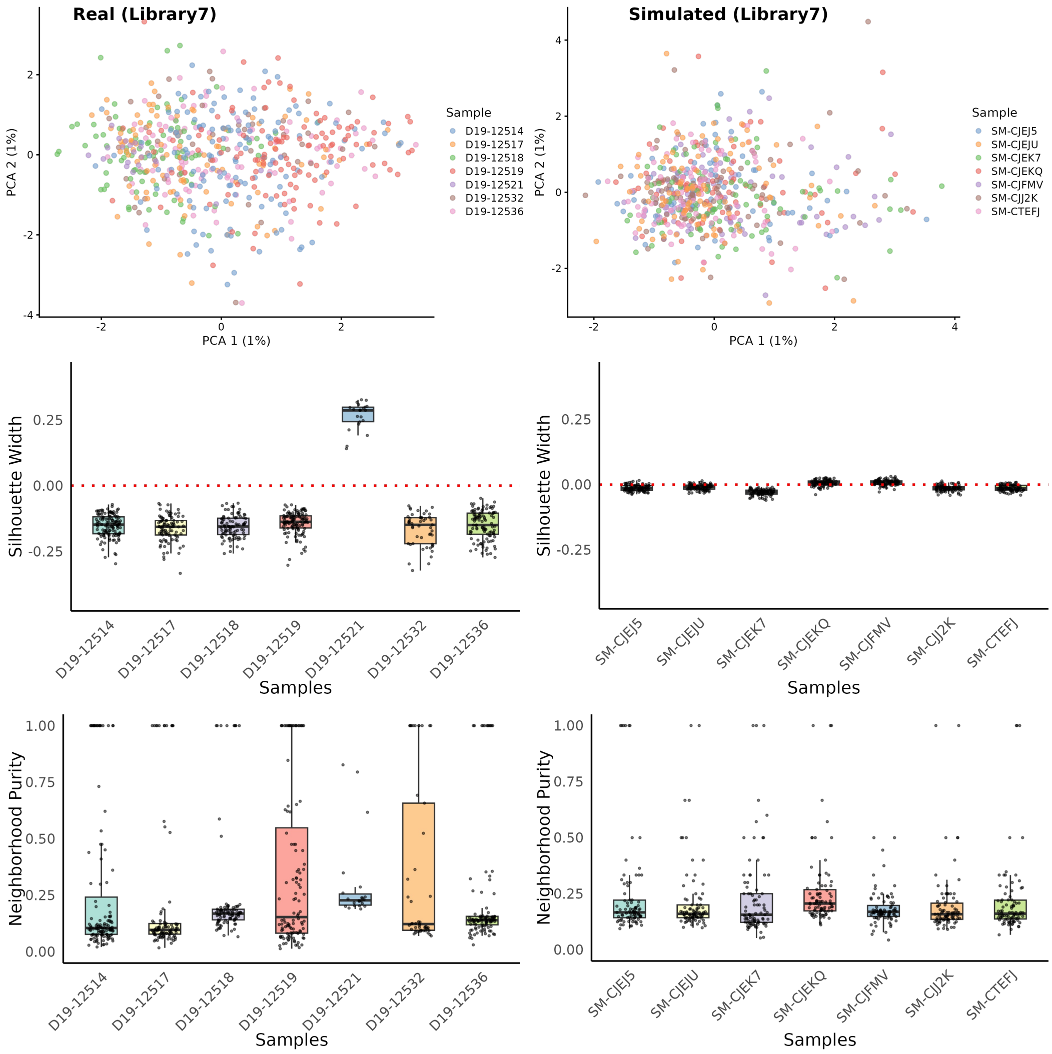

Fig. S6 Quantifying clustering in real and simPIC-simulated data for early AD library 7 with 1693 peaks and 604 cells. (Top row) PCA plot of real and simulated data, coloured by individual, showing the preservation of individual-specific structure. (Middle row) Silhouette width comparison between real and simulated data, assessing cluster compactness. (Bottom row) Neighbourhood purity analysis, evaluating local structure consistency across real and simulated datasets.

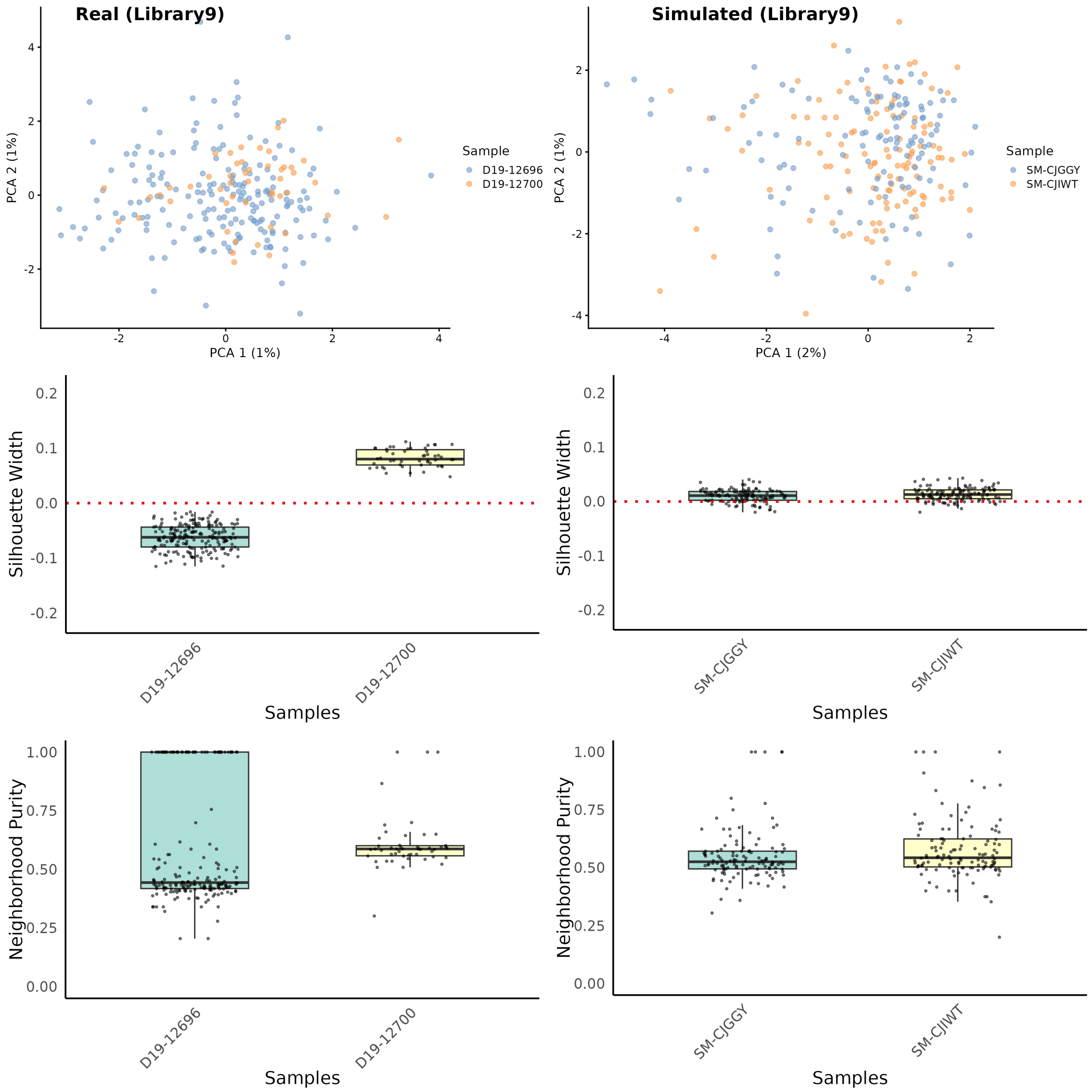

Fig. S7 Quantifying clustering in real and simPIC-simulated data for early AD library 9 with 1693 peaks and 244 cells. (Top row) PCA plot of real and simulated data, coloured by individual, showing the preservation of individual-specific structure. (Middle row) Silhouette width comparison between real and simulated data, assessing cluster compactness. (Bottom row) Neighbourhood purity analysis, evaluating local structure consistency across real and simulated datasets.

The below figures are for nonAD samples

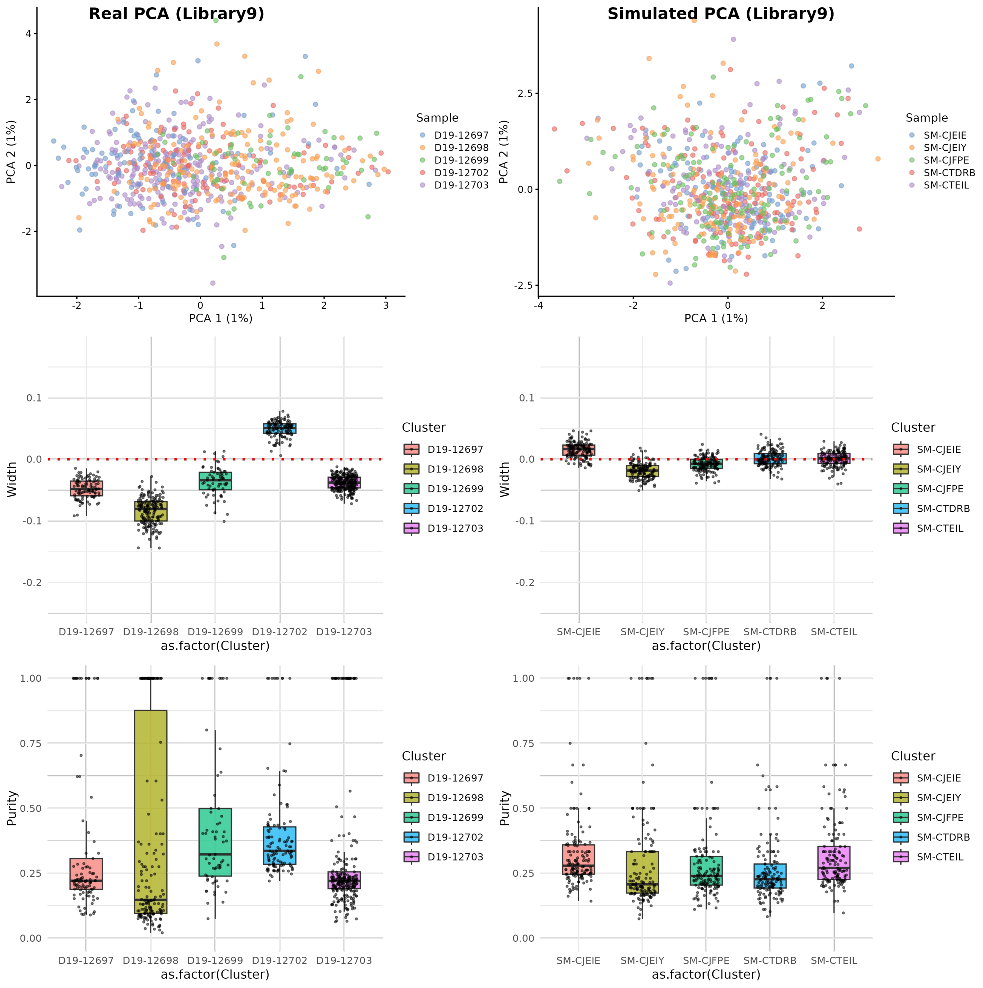

Fig. S8 Quantifying clustering in real and simPIC-simulated data for non AD library 9 with 1693 peaks and 710 cells. (Top row) PCA plot of real and simulated data, coloured by individual, showing the preservation of individual-specific structure. (Middle row) Silhouette width comparison between real and simulated data, assessing cluster compactness. (Bottom row) Neighbourhood purity analysis, evaluating local structure consistency across real and simulated datasets.

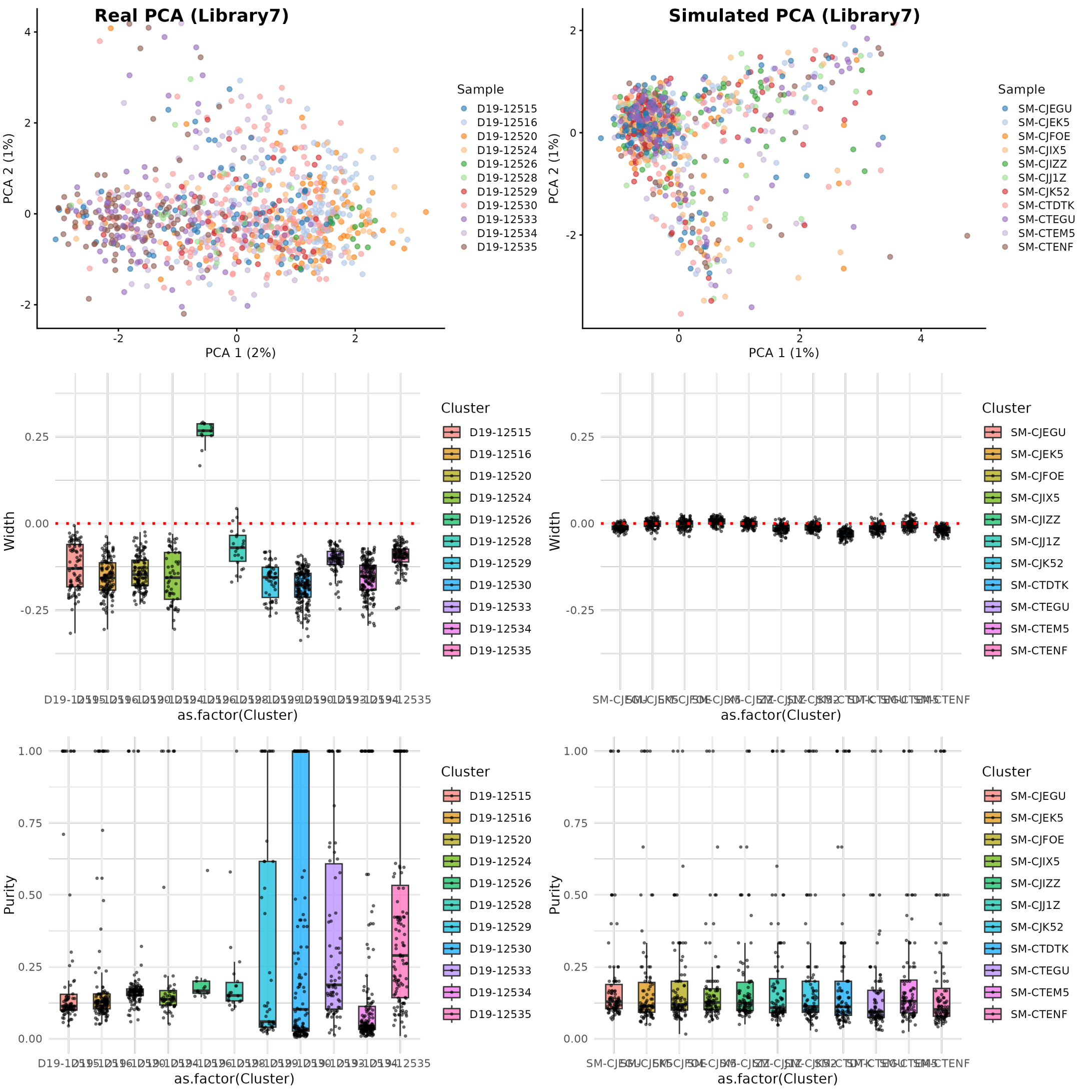

Fig. S9 Quantifying clustering in real and simPIC-simulated data for non AD library 7 with 1693 peaks and 1012 cells. (Top row) PCA plot of real and simulated data, coloured by individual, showing the preservation of individual-specific structure. (Middle row) Silhouette width comparison between real and simulated data, assessing cluster compactness. (Bottom row) Neighbourhood purity analysis, evaluating local structure consistency across real and simulated datasets.

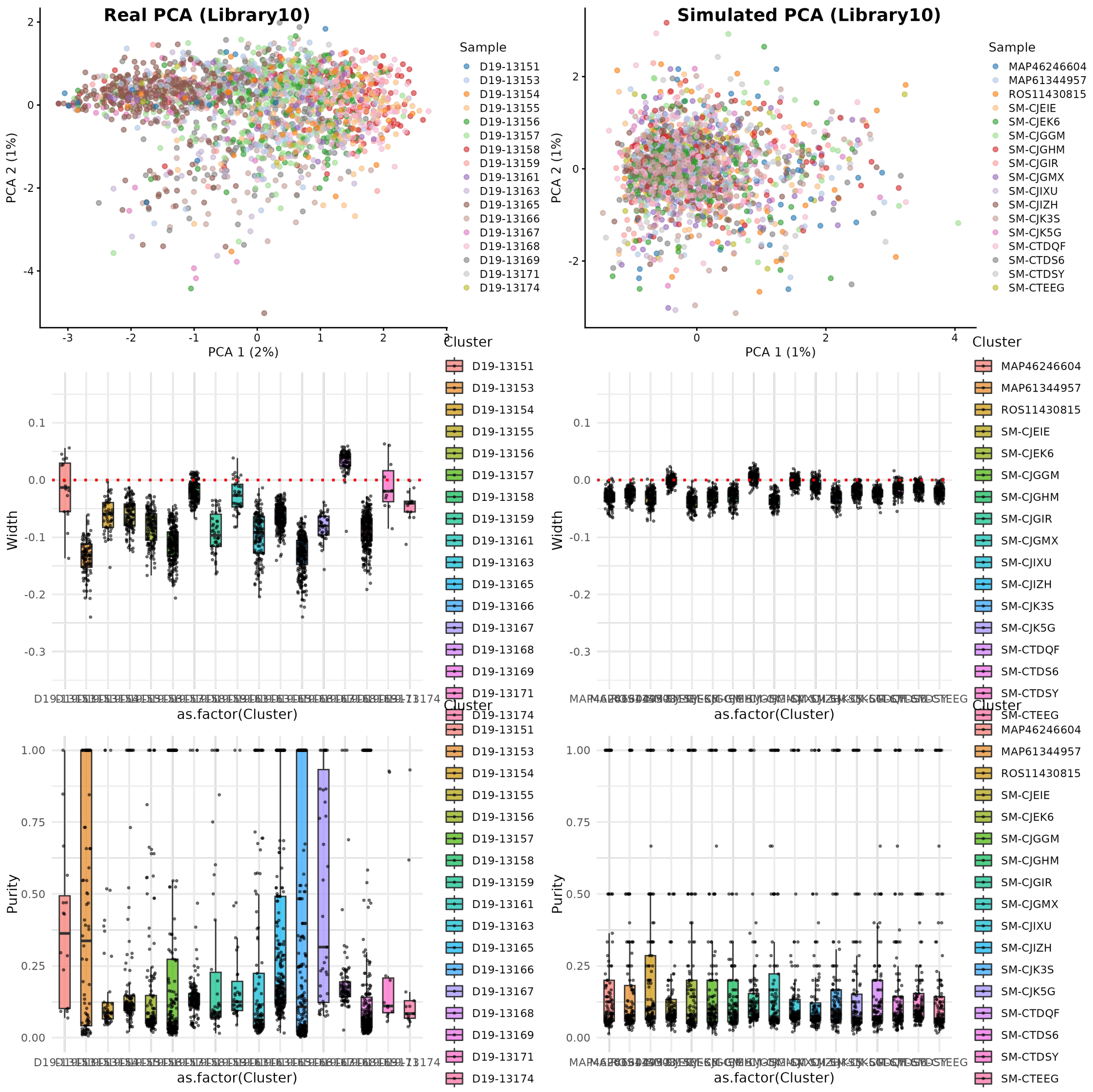

Fig. S10 Quantifying clustering in real and simPIC-simulated data for non AD library 10 with 1693 peaks and 2057 cells. (Top row) PCA plot of real and simulated data, coloured by individual, showing the preservation of individual-specific structure. (Middle row) Silhouette width comparison between real and simulated data, assessing cluster compactness. (Bottom row) Neighbourhood purity analysis, evaluating local structure consistency across real and simulated datasets.

References

[1] F. Zhao, X. Ma, B. Yao, Q. Lu, L. Chen, scada: A novel statistical method for differential analysis of single-cell chromatin accessibility sequencing data. PLOS Computational Biology **20**(8), e1011854 (2024)

[2] H. Zhang, R.M. Mulqueen, N. Iannuzo, D.O. Farrera, F. Polverino, J.J. Galligan, J.G. Ledford, A.C. Adey, D.A. Cusanovich, txci-atac-seq: a massive-scale single-cell technique to profile chromatin accessibility. Genome biology **25**(1), 78 (2024)

[3] M.D. Robinson, D.J. McCarthy, G.K. Smyth, edger: a bioconductor package for differential expression analysis of digital gene expression data. bioinformatics **26**(1), 139–140 (2010)

[4] L. Zappia, B. Phipson, A. Oshlack, Splatter: simulation of single-cell rna sequencing data. Genome biology **18**(1), 174 (2017)
